# AMDE-1 Is a Dual Function Chemical for Autophagy Activation and Inhibition

**DOI:** 10.1371/journal.pone.0122083

**Published:** 2015-04-20

**Authors:** Min Li, Zuolong Yang, Laura L. Vollmer, Ying Gao, Yuanyuan Fu, Cui Liu, Xiaoyun Chen, Peiqing Liu, Andreas Vogt, Xiao-Ming Yin

**Affiliations:** 1 School of Pharmaceutical Sciences, Sun Yat-Sen University, Guangzhou, Guangdong, China; 2 Department of Pathology and Laboratory Medicine, Indiana University School of Medicine, Indianapolis, Indiana, United States of America; 3 University of Pittsburgh Drug Discovery Institute, Pittsburgh, Pennsylvania, United States of America; 4 Department of Computational and Systems Biology, University of Pittsburgh School of Medicine, Pittsburgh, Pennsylvania, United States of America; University of Alabama at Birmingham, UNITED STATES

## Abstract

Autophagy is the process by which cytosolic components and organelles are delivered to the lysosome for degradation. Autophagy plays important roles in cellular homeostasis and disease pathogenesis. Small chemical molecules that can modulate autophagy activity may have pharmacological value for treating diseases. Using a GFP-LC3-based high content screening assay we identified a novel chemical that is able to modulate autophagy at both initiation and degradation levels. This molecule, termed as Autophagy Modulator with Dual Effect-1 (AMDE-1), triggered autophagy in an Atg5-dependent manner, recruiting Atg16 to the pre-autophagosomal site and causing LC3 lipidation. AMDE-1 induced autophagy through the activation of AMPK, which inactivated mTORC1 and activated ULK1. AMDE-1did not affect MAP kinase, JNK or oxidative stress signaling for autophagy induction. Surprisingly, treatment with AMDE-1 resulted in impairment in autophagic flux and inhibition of long-lived protein degradation. This inhibition was correlated with a reduction in lysosomal degradation capacity but not with autophagosome-lysosome fusion. Further analysis indicated that AMDE-1 caused a reduction in lysosome acidity and lysosomal proteolytic activity, suggesting that it suppressed general lysosome function. AMDE-1 thus also impaired endocytosis-mediated EGF receptor degradation. The dual effects of AMDE-1 on autophagy induction and lysosomal degradation suggested that its net effect would likely lead to autophagic stress and lysosome dysfunction, and therefore cell death. Indeed, AMDE-1 triggered necroptosis and was preferentially cytotoxic to cancer cells. In conclusion, this study identified a new class of autophagy modulators with dual effects, which can be explored for potential uses in cancer therapy.

## Introduction

Autophagy is a universal, dynamic degradation process that takes place in all eukaryotic cells and contributes to the turnover and rejuvenation of cellular components via the lysosome pathway [1]. Autophagy plays significant roles in human diseases including cancer, neurodegenerative diseases, infectious and inflammatory diseases [2]. Because of the potential importance in regulating autophagy for therapeutic manipulations, there is great demand for potent modulators of the autophagic pathway.

Recently developed screening assays for small molecule modulators of autophagy have employed a variety of readouts [3,4,5]. The most commonly used parameter is the lipidation of LC3, a well-established autophagosome marker [6,7,8,9]. In particular, cell-based high-content screening assays examine the translocation of GFP-LC3 from the cytosol to autophagic membranes as a result of LC3 lipidation, which causes the appearance of GFP signals in punctate structures. A number of chemicals have been found that affect the extent of LC3 lipidation as a result of enhanced autophagy activation or decreased autophagic degradation [6,7,8,9].

Autophagy inducers are those chemicals that can activate autophagy either via suppressing mTORC1, such as rapamycin, or via mechanisms not related to mTORC1 suppression, such as carbamazepine (CBZ) [10,11]. Autophagy inhibitors can work by inhibiting upstream autophagy machinery, such as the Class III phosphatidylinositol 3-kinase (PIK3C3) and Beclin 1. 3-methyladenine (3-MA) is a PIK3C3 inhibitor that suppresses autophagy [12], while spautin-1 inhibits autophagy by promoting Beclin 1 degradation [13]. Lysosome inhibitors, such as chloroquine (CQ) and bafilomycin A_1_ can also inhibit autophagy at the degradation stage [8]. Bafilomycin A_1_ may also block autophagosome-lysosome fusion [14].

Chemical modulators have been successfully used in basic research, although their use in the clinic is just being explored [10,11]. One of the potential uses of autophagy enhancers is in promoting autophagic degradation of misfolded or aggregated proteins, such as mutant huntingtin [15] or mutant alpha1-antitrypsin [16], whereas autophagy inhibitors could be potentially used in cancer therapy [17]. Autophagy is an important biological process in cancer. Autophagy has a suppressive effect against tumorigenesis at the initiation stage but cancer cells could utilize autophagy for cytoprotection once the tumor is established [18]. Autophagy provides a cytoprotective mechanism for cancer cells exposed to cytotoxic therapy. A combined use of CQ with some of the routinely used chemotherapeutic agents proves to be quite valuable in overcoming autophagy-mediated cytoprotection in cancer therapy [17,19].

In this study, we used a high-content screening assay based on GFP-LC3 translocation to identify potential autophagy modulators. Surprisingly, among the several potential modulators we found one chemical that possessed a dual effect on both autophagy activation and autophagy-mediated degradation. This compound, named as AMDE-1 for Autophagy Modulator with Dual Effect-1, activated autophagy by the AMPK-mTORC1-ULK1 pathway and at the same time inhibited autophagy-mediated degradation by causing lysosome dysfunction. We also found that AMDE-1 had potent cytotoxic effects preferentially against cancer cells, suggesting its potential applications in cancer therapy.

## Materials and Methods

### Antibodies and chemicals

Antibodies against AMPK, phosphor-AMPK (Thr172), UKL1, phosphor-ULK1 (Ser555), phosphor-ULK1 (Ser757), p70S6K1, phospho-p70S6K1 (Thr389), S6, phospho-S6 (Ser235/236), 4E-BP1, phospho-4E-BP1 (Thr37/46), cathepsin D, and EGF receptor were obtained from Cell Signaling Technology (Danvers, MA). Antibodies against SQSTM1/ p62, LC3B and Atg16L were purchased from MBL International (Woburn, MA). Anti-cathepsin B and GFP antibodies were obtained from Santa Cruz Biotechnology (Santa Cruz, CA); and anti-LAMP2 was obtained from Developmental Studies Hybridoma Bank (Iowa City, IA). Secondary antibodies conjugated with Alexa Fluor-488, Cy3 or horseradish peroxidase were purchased from Life Technologies (Carlsbad, CA) and Jackson Immuno Research Laboratories (West Grove, PA), respectively.

AMDE-1 (2-[5,7-bis(trifluoromethyl)[1,8]naphthyridin-2-yl]-2-(3-chlorophenyl)acetonitrile was repurchased from Key Organics (Bedford, MA) with a purity of >90%. Ammonium chloride (AC), chloroquine (CQ), acridine orange (AO), 3-methyladenine (3-MA and human EGF were purchased from Sigma-Aldrich (St Louis, MO). Bafilomycin A_1_ (Baf) and rapamycin (Rap) were obtained from LC Laboratories (Woburn, MA). E64d and pepstatin A were obtained from A. G. Scientific (San Diego, CA). Z-VAD-FMK and necrostatin-1 were from SelleckChem (Houston, TX). Other reagents used were the cocktail protease/phosphatase inhibitors (Roche, Indianapolis, IN), Lipofectamine 2000 and LysoTracker Red (LTR, Life Technologies, Carlsbad, CA) and L-[^14^C]-valine (PerkinElmer, Waltham, MA).

### Cell culture

Wild type and Atg5-deficient MEFs, HEK293, A549, U251, HL60, HCT116 and CCD-18Co cells have been described before [8]. Cells were cultured at 37°C in a humidified air atmosphere with 5% (v/v) of CO2. The complete medium is DMEM (Thermo Scientific, Rockford, IL) with 10% (v/v) of fetal bovine serum (Gibco, Grand Island, NY) and other standard supplements. EBSS with no serum and other supplements was used as starvation medium.

### High content screening assay

The high content screening assay for autophagy modulators was based on the change in GFP-LC3 punctation. This assay was developed, optimized, and conducted at the Pittsburgh Molecular Libraries Screening Center (PMLSC) according to universally accepted HTS guidelines [20]. The optimized assay used 1 μM thapsigargin as positive control, showed DMSO tolerance up to 1.4%, and in multi-day variability assessments returned signal-to-background ratios and Z-factors of 10.7/0.47, 16.3/0.30, and 5.4/0.35 on days 1, 2, and 3, respectively. The results of the screen and a detailed protocol can be found on Pubchem (BioAssay: AID 463193; https://pubchem.ncbi.nlm.nih.gov/assay/assay.cgi?aid=463193). Briefly, MEFs stably expressing GFP-LC3 were seeded in collagen-coated 384 well microplates (Falcon Biocoat) and incubated overnight in 5% CO_2_ at 37°C. The density of the culture was 5,000 per well in 40 μL of complete medium (DMEM with 10% FCS). The chemical library used in this screen was the NIH Molecular Libraries Small Molecule Collection (https://mlsmr.evotec.com/MLSMR_HomePage/index.html). The library was stored in DMSO and diluted in complete medium before transfer to the assay plates. The final concentration of compounds was 10 μM and that of DMSO was 1%.

After incubation for 18 hours the cells were fixed with 10 μL of fixation solution (10.7% formaldehyde and 26.7 μg/ml Hoechst 33342 in HBSS) for 30 min. Following three washes with PBS, the plates were analyzed on the ArrayScan VTi (Thermo Fisher Cellomics) for autophagosomal GFP-LC3 accumulation. Images were acquired in two channels (DAPI/FITC) using a 10x objective and an Omega XF100 filter set, and were analyzed by an in-house developed imaging algorithm based on the Cellomics Compartmental Analysis Bioapplication (S1 Fig). In brief, a nuclear mask was generated from Hoechst-stained nuclei. An area representative of the cytoplasm was generated by placing a set of concentric rings around the nuclear mask. To exclude perinuclear fluorescence artifacts, the nuclear mask was dilated by three pixels before defining the cytoplasmic region. Autophagosomal membrane-associated GFP-LC3 was detected as fluorescent punctate objects (puncta, or spots) that exceeded a threshold defined by untreated cells and that were located exclusively in the cytoplasmic area.

A selection threshold was then set using DMSO-treated cells to calculate the percentage of cells positive for GFP-LC3 puncta formation. This parameter had greater discriminatory power than the number of cells containing puncta. Based on the results from the variability assessment studies, we scored positive agents as those that increased the percentage of GFP-LC3 puncta positive cells with a z-score of greater than 3 (based on plate average). To triage fluorescence and toxicity artifacts, compounds that showed overall GFP-intensity in the cytoplasm greater than 1500 and caused more than 80% cell loss were eliminated.

### Immunoassay

For immunoblot assay, proteins were separated by SDS-PAGE and transferred to PVDF membranes (Millipore, Billerica, MA). Primary antibodies were used, followed by HRP-conjugated secondary antibodies. Specific proteins were detected using Immobilon western chemiluminescence HRP substrate (EMD Millipore, Billerica, MA). Densitometry was performed with a Kodak Image Station 4000 and the companion software (Carestream Health, Inc. Rochester, NY).

Immunofluorescence staining was carried out as described previously [8]. Briefly, cells were grown on glass cover slips and were fixed in 4% formaldehyde followed by permeabilization and blocking in PBS containing 2% bovine serum albumin (BSA). Primary and Alexa488-conjugated secondary antibodies were applied sequentially. Samples were mounted on glass slides after nuclei staining. Fluorescence images were taken using a Nikon Eclipse TE200 Epi-fluorescence microscope with NIS-Elements AR3.2 software. At least 50 cells per experimental condition were analyzed for quantification.

### Long-lived protein degradation assay

Long-lived protein degradation assay was performed as previously described [21]. Briefly, cells were cultured in DMEM in 24-well plates. L-[^14^C]-valine was added to a final concentration of 0.2 μCi/ml to label intracellular proteins. Cells were seeded for 18 h before changing to fresh medium with 10% cold L-valine for another hour to deplete labeled short-lived proteins. The cells were then incubated in EBSS or DMEM (plus 0.1% of BSA and 10 mM valine) with or without the testing chemicals for an additional 6 or 16 hours. The culture medium was then recovered, from which the degraded long-lived proteins were measured via liquid scintillation (Beckman LS 6000).

### Analysis of lysosome function

Lysosomal acidity was examined by incubating cells with LTR (50 nM) or AO (1 μg/ml) for 20 to 30 minutes at 37°C in complete medium followed by treatment. The fluorescence intensity was then measured by fluorescence microscopy.

The enzymatic activity of cathepsins was measured as previously described [8]. Briefly, after experimental treatment cells were suspended in hypotonic buffer (40 mM KCl, 5 mM MgCl_2_, 2 mM EGTA, 10 mM HEPES, pH 7.5) for 30 min on ice. Cells were homogenized by shearing through a 28.5 G needle for 30 times and the lysosome-enriched fraction (P10) was isolated by differential centrifugation, which was washed in an isotonic buffer (150 mM NaCl, 5 mM MgCl_2_, 2 mM EGTA, 10 mM HEPES, pH 7.5) and dissolved in native lysis buffer (1% Triton X-100, 150 mM NaCl, 50 mM Tris-HCl, pH 7.5) for activity analysis. The substrates Z-FR-AMC and Mca-Gly-Lys-Pro-Ile-Leu-Phe-Arg-Leu-Lys(Dnp)-D-Arg-NH_2_ (Enzo Life Sciences, Farmingdale, NY) was used for cathepsin B and cathepsin D assay, respectively.

To determine the lysosomal degradation capacity, cells were incubated with 10 μg/ml of self-quenched bodipy-conjugated BSA (DQ-BSA Red) for 1 h at 37°C. Following the wash new medium was added with or without the designated chemicals for 4 to 6 h. Degradation capacity was measured by monitoring the appearance of red fluorescent dots due to the degradation of DQ-BSA-Red, which was then quantified.

### Analysis of EGF receptor degradation

HeLa cells were starved in serum-free medium DMEM for 1.5 h and were either mock-treated as controls or incubated with EGF (200 ng/ml) with cycloheximide (20 μg/ml) and other designated chemicals in 37°C for 0–4.5 hours. Cells were then washed with cold PBS and immediately lysed. Lysates were analyzed by immunoblotting assay using an anti-EGF receptor antibody.

### Cell viability assay

Cells were seeded at 5×10^3^ per well in 96-well plates and incubated overnight with the designated chemicals for 24–48 h. Cell viability was measured by adding 10% MTS (CellTiter 96, Promega Corporation, Madison, USA) for additional 2 h. The absorbance was measured at 490 nm with a spectrophotometer (Infinite M1000, Tecan). Cell death was calculated from three independent experiments and normalized to the absorbance of wells containing medium only with no cells (100%) and that of wells containing untreated cells (0%). Dose responses were fitted with appropriate regression models using Microsoft Excel’s Trendline tool. EC_50_ doses were calculated using the regression equations, where the 50% response point was defined using the full range of response (0–100%) for the absolute EC_50_ values and using the detected response range (analyte-specific) for the relative EC_50_ values.

### Statistical analysis

All data are presented as the mean ± SD from at least three separate experiments. The p values were determined by two-tailed Student’s *t*-test. P < 0.05 was considered as being significant.

## Results

### High Throughput screening for modulators of GFP-LC3 punctation

Using murine embryonic fibroblasts (MEFs) that express GFP-LC3 we conducted a high content screening for potential chemical modulators of autophagy based on changes in GFP-LC3 translocation to autophagosomal membranes (S1 Fig). A total of 1,963 chemicals from the library of 196,160 compounds were found to be positive for upregulating GFP-LC3 puncta based on the criteria described in the Methods section. This yielded a hit rate of 1%. The results of this screen, including experimental details, have been deposited to PubChem (AID: 463193).

One thousand seven hundred thirty (1730) compounds were available for cherry-picking and were retested at the single-dose of 10 μM using the same image analysis algorithm used in the primary screen. Seven hundred twenty one (721) agents increased the number of cells with puncta by more than three standard deviations over the negative control (confirmation rate: 42%), and were subjected to three independent ten-point dose dependence trials (20 nM to 10 μM). Average EC50s were calculated from all three experiments; EC50 values that were artifacts of curve fitting were eliminated. The remaining compounds were visually examined for the presence of puncta at 10 μM. Compounds were eliminated if there was a presence of fluorescent artifacts, if the compound precipitated out of solution, or if the compound did not demonstrate a clear punctate phenotype. Fifty six agents had complete dose responses with EC50 values below 10 μM (S1 Table). Structural similarity analysis using Leadscope Enterprise 2.4.6–1 Chemical Structure analysis software revealed nine clusters for 24 agents and 32 singletons. All 56 agents were subsequently re-examined in MEFs stably expressing GFP-LC3 and their ability to induce GFP-LC3 puncta was quantified manually. This allowed a rigorous visual confirmation and the percentage of cells with 5 or spots per cells were calculated (S2 Table). Furthermore, the specificity of their effects was evaluated using MEFs lacking Atg5, an essential gene for autophagy. Finally, we used Western blot analysis to examine the lipidated form of GFP-LC3 as an alternative way to confirm the effects of the top hits (S2 Table). The multiple secondary assays ensured that the effect of these chemical compounds in modulating GFP-LC3 puncta was authentic and repeatable. Several of these compounds were found to be non-specific (e.g., SID 14741249, SID 17408254 and SID 14733320) or to cause cellular destruction (e.g.,SID 14729169, SID17507447 and SID 24826599).

### AMDE-1 is a novel compound that can activate autophagy

**Fig 1:**
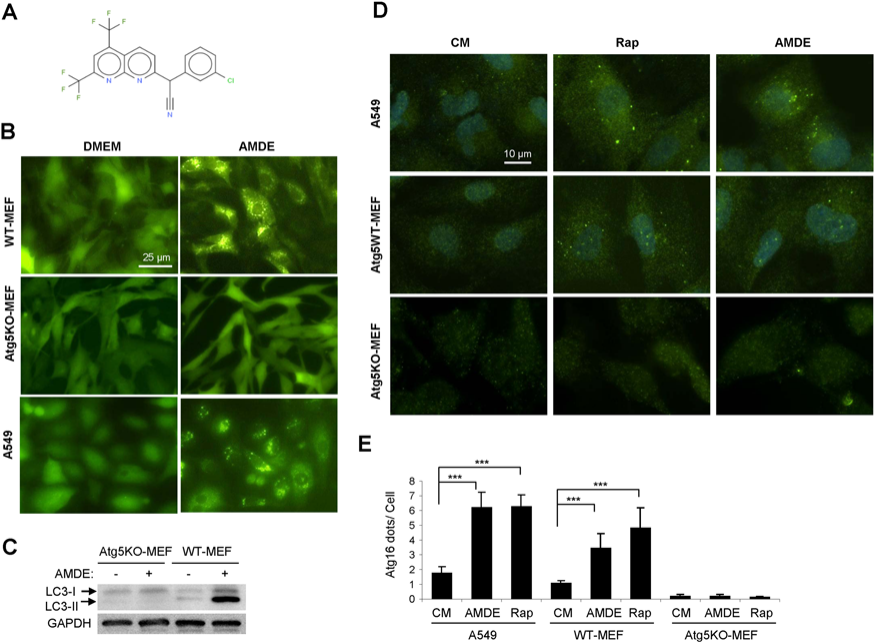
AMDE-1 can induce autophagy. **(A)** The molecular structure of AMDE-1. **(B-C**) A549, wild type MEFs and Atg5 KO MEFs expressing GFP-LC3 were treated with or without AMDE-1 (10 μM) for 6 h, and then analyzed by GFP-LC3 puncta formation (B) or by immunoblotting (C). (**D-E**) A549, wild type MEFs and Atg5 KO MEFs were treated with 1 μM rapamycin (Rap) or 10 μM AMDE-1 for 6 h, followed by immunostaining with an anti-Atg16L1 antibody (D). Atg16L1 puncta were quantified (E). Values represent means ± SD from three independent experiments. ***: p<0.001. CM: control medium.

Several agents caused rapid and sustained accumulation of autophagosomes in GFP-LC3-MEFs. One of them was brefeldin A (SID 855810), which is a known autophagy inducer [22], thus validating the screen and the secondary assay scheme. The most potent and specific inducer of autophagy was SID 14730495, which caused 96.7% of cells to accumulate GFP-LC3 puncta at the earliest time point (11 hours) and had essentially no activity in Atg5KO-MEF (S2 Table). We thus decided to conduct an in-depth analysis for the mechanism by which it affected autophagy. This compound, which we named Autophagy Modulator with Dual Effect-1 (AMDE-1), has a molecular weight of 415.7 (C_18_H_8_ClF_6_N_3_) (Fig 1A) and its chemical name based on IUPAC (International Union of Pure and Applied Chemistry) nomenclature is 2-[5,7-bis(trifluoromethyl)[1,8]naphthyridin-2-yl]-2-(3-chlorophenyl)acetonitrile.

**Fig 2:**
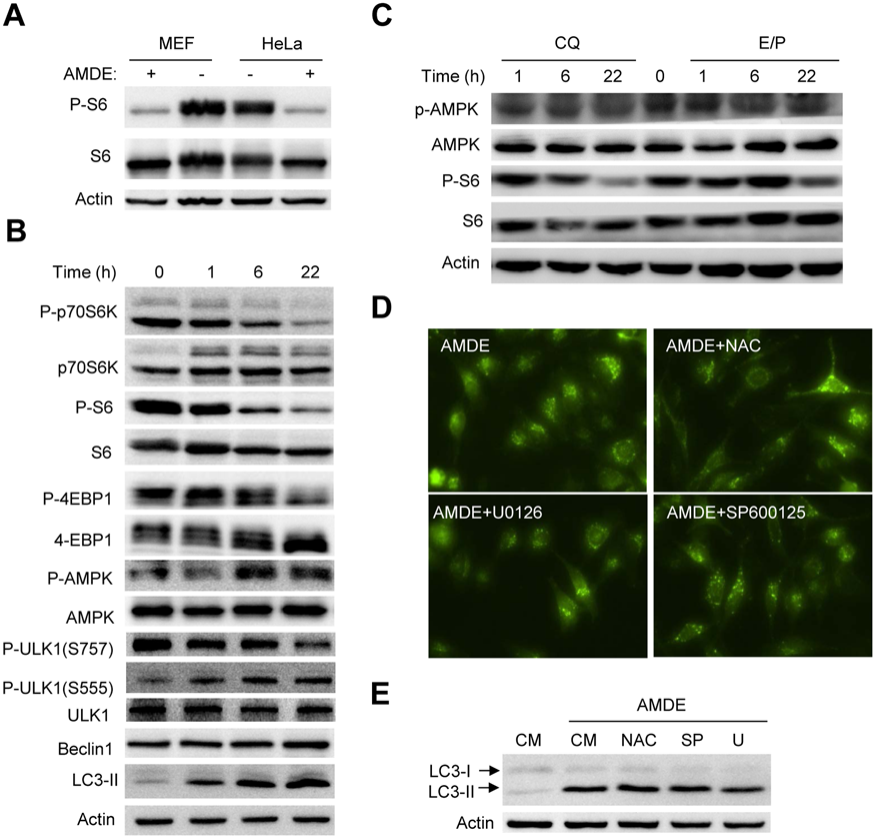
AMDE-1 can induce autophagy through AMPK-mTOR-ULK1 pathway. (**A**) Wild-type MEFs and HeLa cells were treated with or without 10 μM of AMDE-1 for 6 h. Phosphorylation of S6 was detected by immunoblotting assay. (**B-C**) MEFs were treated with 10 μM AMDE-1 (B), or chloroquine (CQ, 40 μM) or E/P (E64D, 25 μM plus pepstatin A, 50 μM) (C) for different times as indicated and analyzed by Western blot. The zero time point represents no treatment. (**D-E**) MEFs expressing GFP-LC3 were treated with AMDE-1 alone or together with N-acetyl cysteine (NAC, 20 μM), U0126 (30 μM), or SP600125 (20 μM) for 6 h. GFP-LC3 puncta formation (D) and endogenous LC3 level were analyzed (E).

As found in our screening assay, AMDE-1 induced GFP-LC3 puncta in the MEFs. This induction was specific to autophagy activation as it was eliminated in cells lacking Atg5, a key autophagy gene (Fig 1B, S2 Table). GFP-LC3 puncta could also be observed in other types of cells such as A549 (Fig 1B, S2 Table, Saos2 (S2 Table), and HEK293 (Fig 2). Consistent with the imaging analysis, lipidation of GFP-LC3 (S2 Table) and of endogenous LC3 was detected by immunoblotting in wild type but not in Atg5-deficient MEFs (Fig 1C). During autophagy initiation, Atg5 is conjugated with Atg12, which is bound to Atg16L1 to form a complex on the pre-autophagosomal membrane that is necessary for the lipidation of LC3 and autophagosome formation [23,24]. Atg5-Atg12-Atg16L1 complex is then detached from the completed LC3-positive autophagosomes, therefore acting as an early autophagy marker independent of later events like fusion with, and degradation in, the lysosome. When stained with an anti-Atg16L1 antibody, both A549 and MEFs treated with AMDE-1 demonstrated the formation of Atg16L1-positive puncta in a pattern (Fig 1D) and at the level (Fig 1E) comparable to that induced by a classic autophagy inducer, rapamycin (Rap). Furthermore Atg16L1 punctation, like LC3 punctation, depends on Atg5. Taken together, these data strongly indicated that AMDE-1 was able to initiate autophagy using the defined autophagy machinery.

### The AMPK-mTOR-ULK1 pathway is involved in AMDE-1 induced autophagy

Small molecules can affect autophagy activation through either mTOR-dependent or mTOR-independent pathway [11]. We examined whether AMDE-1 initiated autophagy by affecting mTOR signaling. Indeed, we found that AMDE-1 suppressed the phosphorylation of S6, a downstream target of mTORC1, in both MEFs and HeLa (Fig 2A). A time-dependent analysis in MEFs indicated that the effect could be detected 1 hour after treatment and was associated with the inhibition of p70S6 kinase and 4E-BP1, both being direct targets of mTORC1 (Fig 2B). AMPK can promote autophagy indirectly by inactivating mTORC1 and directly by activating the ULK1/Atg1 complex [25,26]. We found that in the same time frame as mTORC1 was inhibited phosphorylation of AMPK at Thr172 was increased, indicating that AMDE-1 could inhibit mTORC1 by activating AMPK.

As AMDE-1 had also inhibitory effects on the lysosome (see below), we wondered whether AMPK activation could be triggered by a general suppression of the lysosome function. Treatment of cells with chloroquine, which reduced lysosomal acidity, or with E64D plus pepstatin A, which inhibited the lysosomal proteases, did not lead to AMPK phosphorylation, despite that these chemicals inhibited S6 phosphorylation as reported before [8] (Fig 2C). These results indicate that lysosomal suppression does not necessarily lead to AMPK activation, and that AMDE-1 can activate AMPK, and thus autophagy, independently of lysosome inhibition.

Canonical autophagy is initiated at the ULK1/Atg1 complex, which is evolutionarily conserved from yeast to the mammals [2]. Mammalian ULK1 is inhibited by mTORC1 through the phosphorylation at Ser^757^ whereas it can be activated by AMPK through the phosphorylation at Ser^555^ [25,26]. Indeed, we found that AMDE-1 treatment caused an increased ULK1 phosphorylation at Ser^555^ but a decreased phosphorylation at Ser^757^ in a time-dependent fashion (Fig 2B), supporting the roles of an activated AMPK and an inactivated mTOC1 in AMDE-1-stimulated autophagy activation.

Several other signaling pathways including ERK, JNK and reactive oxygen species (ROS) have been reported to be involved in autophagy activation [27,28]. However, by employing U0126, SP600125 or N-acetyl cysteine (NAC) to respectively suppress these signaling modalities, we did not observe any changes in GFP-LC3 punctation (Fig 2D) or LC3 lipidation (Fig 2E) induced by AMDE-1, suggesting that they were not critically involved in AMDE-1-induced autophagy. Taken together, these findings indicate that AMDE-1 induced autophagy by activating AMPK and inactivating mTORC1.

### AMDE-1 can inhibit autophagic degradation

**Fig 3:**
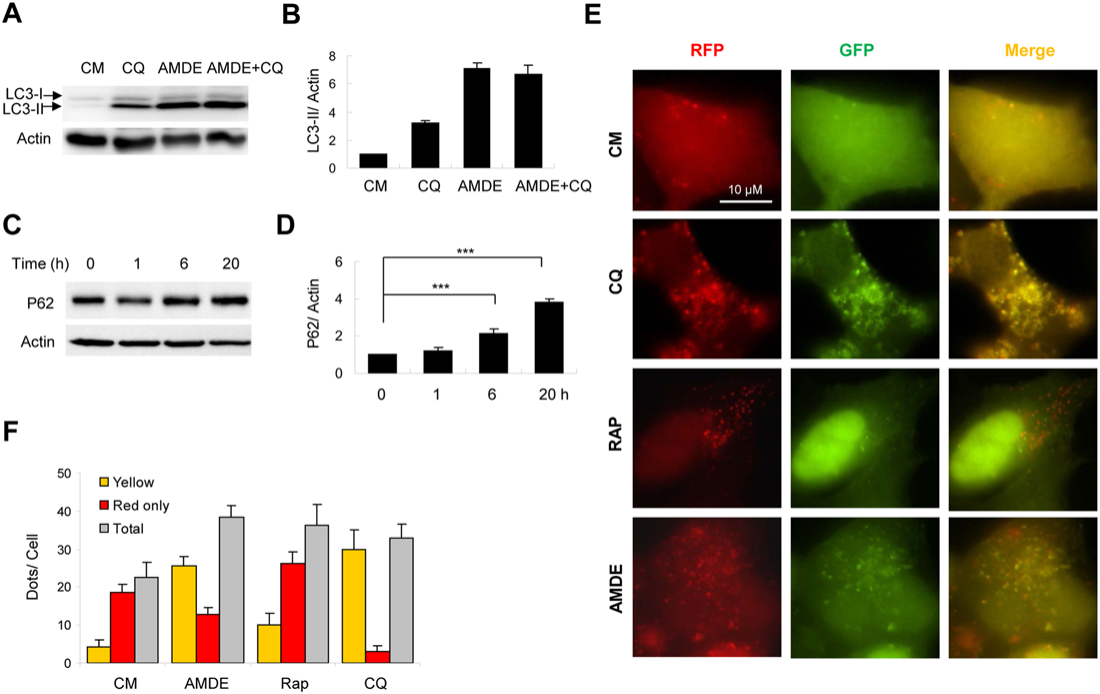
AMDE-1 can inhibit autophagic degradation. (**A-B**) MEFs were treated with or without AMDE-1 (10 μM) and CQ (40 μM) for 20 h. Endogenous LC3 was analyzed by immunoblot assay (A) and densitometry (B). (**C-D**) MEFs were treated with AMDE-1 (10 μM) for 0–20 h as indicated. The level of SQSTM1/p62 was analyzed (C) and quantified (D). (**E-F**) HEK-293A cells expressing GFP-RFP-LC3 were treated with CQ (40 μM), Rap (1 μM) and AMDE-1 (10 μM) for 6 h (E). Puncta showing both green and red fluorescence (indicated as yellow), or showing only the red fluorescence (indicated as red) were quantified (F). (**G-H**) MEFs were incubated in complete medium (CM), or in EBSS with or without AMDE-1 (10 μM), 3-MA (10 mM), CQ (10 μM), Rap (1 μM) or CBZ (50 μM) for 6 h (G) or 16 h (H). The long-lived protein degradation was measured. For panel B, D, F, G and H, values represent means ± SD from three independent experiments. ***: p<0.001; **: p<0.01.

Despite the induction of autophagy by AMDE-1 we noticed that AMDE-1 caused an arrest of autophagy flux. The level of LC3-II in cells treated with AMDE-1 plus chloroquine (CQ) was no higher than that of cells treated with AMDE-1 alone after 20 hours in culture (Fig 3A and 3B). The difference between the two conditions would represent the amount of LC3-positive autophagosomes being degraded through the lysosome. Thus the results suggested that AMDE-1 treatment did not result in autophagic flux. Consistent with this notion, the level of SQSTM1/p62, a sensitive autophagic substrate, increased over time in the presence of AMDE-1 (Fig 3C and 3D). Interestingly, the accumulation of p62 could be detected at 6 hours after treatment, suggesting that the interference could occur early on.

To assess autophagy flux at an early time, we measured the fluorescence change of a tandem RFP-GFP-LC3 construct, which offered a more sensitive and dynamic assay [29]. The normal autophagy process will lead to quenching of GFP once the autophagosome carrying RFP-GFP-LC3 reaches the acidic environment of the lysosome, but the red fluorescence of RFP will not be affected. Thus if the autophagosome is degraded in the lysosome, only red fluorescence will be detected. In contrast, if the autophagosome is not degraded in the lysosome, both RFP and GFP will remain, giving rise to a yellow fluorescence. As anticipated, in HEK293 cells expressing this construct treatment with rapamycin resulted in an increase of total puncta as well as the red fluorescence puncta (Fig 3E and 3F), indicating autophagy activation and the degradation of the labeled autophagosome. In contrast, CQ treatment resulted in increased yellow puncta but decreased red-only puncta, consistent with its effect to inhibit the lysosome (Fig 3E and 3F). The AMDE-1 treatment for 6 hours presented a pattern similar to that of CQ treatment, suggesting a reduced degradation of autophagosomes.

To determine the functional impact of AMDE-1 on autophagy flux, we examined the long-lived protein degradation, which is considered to be the gold standard for assessing autophagy function [30]. Cells incubated in the starvation medium (EBSS) for 6 hours increased long-lived protein degradation, which was inhibited by 3-methyl adenine (3-MA) and CQ, which inhibit autophagy at the initiation and degradation stage, respectively (Fig 3G). The autophagy stimulator, carbamazepine (CBZ), could slightly further increase EBSS-induced long-lived protein degradation. However, AMDE-1 suppressed the degradation as CQ did. Furthermore, AMDE-1 inhibited long-lived protein degradation induced by rapamycin or CBZ in a 16-hour culture (Fig 3H). These data indicated that AMDE-1 plays a dual role in regulating autophagy by initiating autophagy but also by blocking autophagic degradation.

### AMDE-1 can suppress lysosomal degradation

**Fig 4:**
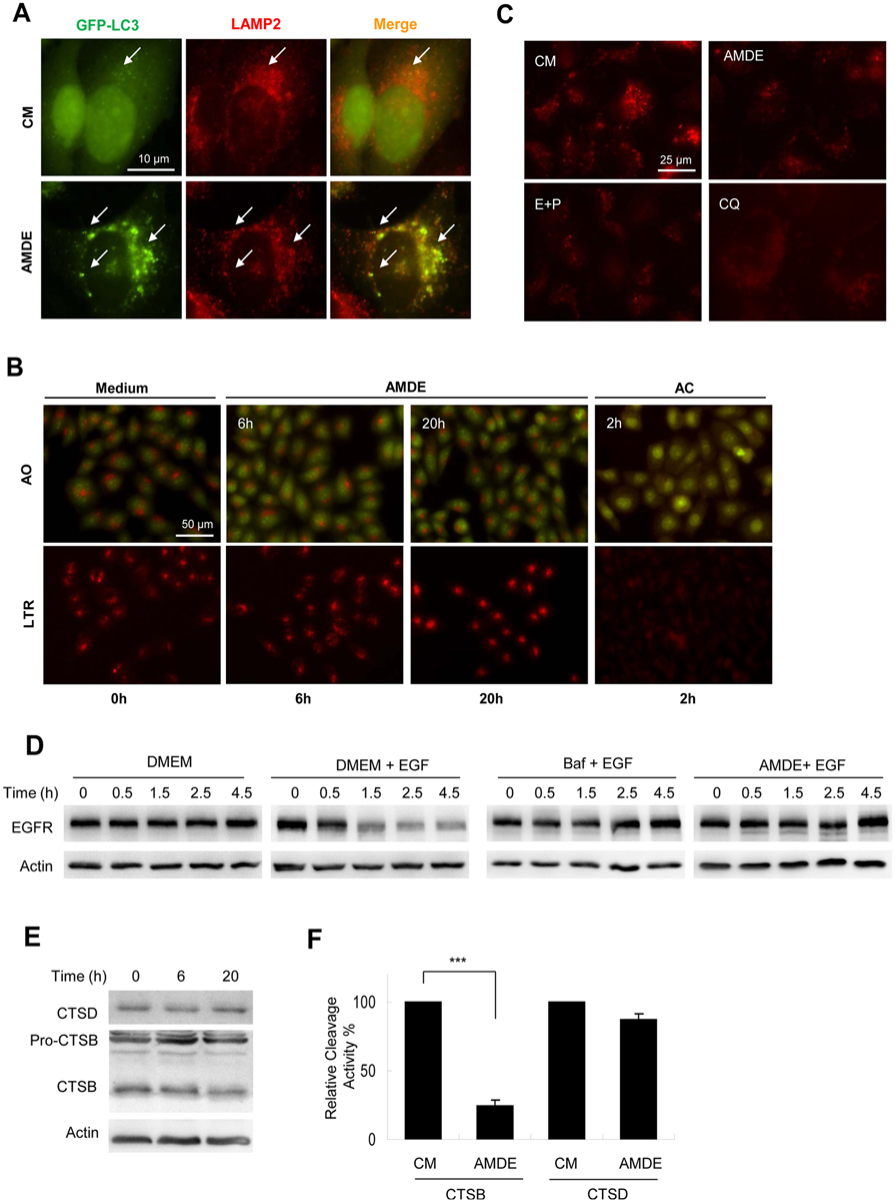
AMDE-1 can block lysosome degradation. (**A**) GFP-LC3-expressing MEFs were treated with AMDE-1 (10 μM) for 6 h, followed by immunostaining with anti-LAMP2. Arrows indicated colocalized GFP-LC3 (green) and LAMP2 (red) puncta. (**B**) HeLa cells were treated with AMDE-1 (10 μM) for 6 or 20 h or with ammonium chloride (AC, 20 mM) for 2 hr. as indicated, followed by staining with acridine orange (AO, 1 μg/ml) or Lyso Tracker Red (LTR, 50 ng/ml) for 30 min. (**C**) HeLa cells were pre-incubated with self-quenched bodipy-conjugated BSA (DQ-BSA, 10 μg/ml) for 1 h and then treated with AMDE-1 (10 μM), E64D (25 μM) plus pepstatin A (50 μM) (E+P) or CQ (40 μM) for 6 h. (**D**) HeLa cells were starved in DMEM for 1.5 h and incubated with or without EGF (200 ng/ml) together with Baf (0.5 μM) or AMDE-1 (10 μM) for 0–4.5 h. EGFR level was detected by immunoblot. (**E**) Hela cells were treated with AMDE-1 (10 μM) for 0–20 h. The lysosome-enriched fraction was analyzed for the expression of cathepsin D (CTSD) and cathepsin B (CTSB). (F) Cells were treated with or without AMDE-1 for 20 hrs, and the activity of cathepsin B and cathepsin D at 20 h was analyzed using the lysosome-enriched fraction. Cathepsin activities were standardized to that of the untreated sample, which was set to 100%. Values represent means ± SD from three independent experiments. ***: p<0.001.

To elucidate how AMDE-1 might inhibit autophagy degradation we first examined the colocalization of AMDE-1-induced GFP-LC3 puncta with the lysosomes as detected by the anti-LAMP2 antibody (Fig 4A). We observed a significant level of colocalization between the two signals, implying that AMDE-1 did not block the fusion between the autophagosome and the lysosome. We then assessed whether AMDE-1 could affect the lysosome acidity. We used two sensitive pH probes that can accumulate in the lysosome. AO is a lysosomotropic metachromatic fluorochrome, and presents green fluorescence in the cytosol but red fluorescence when it is accumulated in the acidic compartment due to its being highly protonated [31]. LysoTracker Red (LTR) manifests red fluorescence in a pH-dependent manner in the lysosome. As seen in Fig 4B, while ammonium chloride (AC) neutralized the pH of the lysosome, resulting in the disappearance of the red fluorescence of both AO and LTR, AMDE-1 had no effect on the fluorescence intensity of the two probes. Thus AMDE-1 did not seem to affect the acidity of the lysosome.

However, we did find that AMDE-1 inhibited lysosome-specific degradation with the DQ-BSA assay. When DQ-BSA is degraded under normal lysosomal conditions, it releases red fluorescence in the lysosome. Treatment of AMDE-1, as well as CQ and E64d plus pepstatin A, caused a significant reduction of DQ-BSA-related red fluorescence (Fig 4C). This result suggested that AMDE-1 might have a general inhibitory effect on the lysosome’s degradation capacity, not just limited to autophagic degradation. To examine this possibility, we examined whether AMDE-1 could affect the degradation of EGF receptor (EGFR) in the lysosome. EGFR was endocytosed upon the addition of EGF to the cell. The degradation of EGFR was monitored by immunoblotting at different times after the addition of EGF (Fig 4D). Degradation of EGFR in normal condition was apparent at 2 hours after EGF addition. AMDE-1, like bafilomycin A_1_ (Baf), a classical lysosome inhibitor, was able to inhibit EGFR degradation in this assay.

To examine how AMDE-1 might affect the lysosomal degradation we isolated the lysosome fraction from AMDE-1-treated cells by differential centrifugation. While the protein level of cathepsin D (CTSD) had not changed, the level of matured cathepsin B (CTSB) was decreased (Fig 4E). Consistently, the enzymatic activity of CTSB was significantly reduced while that of CTSD only decreased slightly (Fig 4F). Taken together, these results indicated that AMDE-1 could inhibit the function of the lysosome by affecting the maturation and activity of selected proteases. This effect led to the inhibition of both autophagic and non-autophagic degradation in the lysosome.

### AMDE-1 is a potent cytotoxic agent for cancer cells

**Fig 5:**
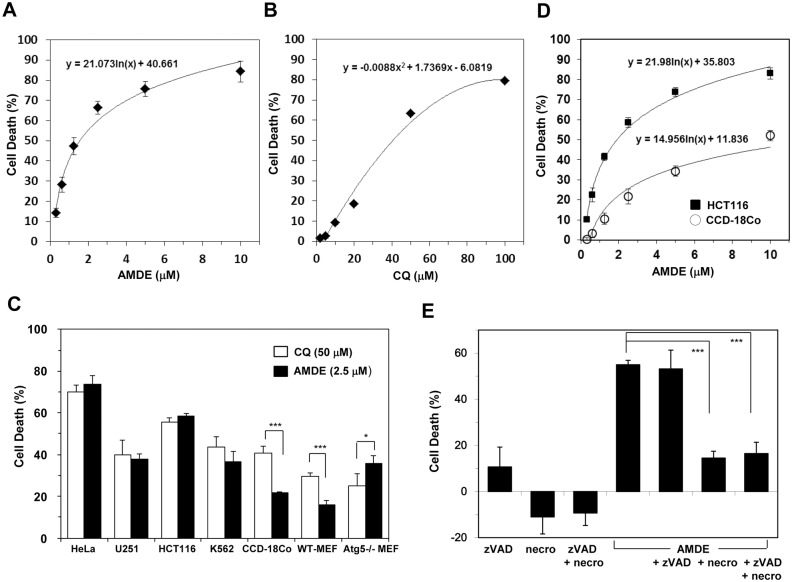
AMDE-1 is a potent cytotoxic agent. (**A-B**) HeLa cells were treated with AMDE (A) or CQ (B) at different doses for 48 hours. Cell death was measured. The dose responses were fitted with a logarithmic regression (A) or a second order polynomial regression (B) algorithm, respectively. (**C)** Cells were treated with CQ (50 μM) or AMDE (2.5 μM) for 48 hours. Cell death was then measured. (**D**) HCT116 and CCD-18Co cells were treated with AMDE at different concentrations for 48 hours. Cell death was measured. The dose response was fitted with a logarithmic regression algorithm. (**E**) HCT16 was treated with or without AMDE-1 (2.5 μM), zVAD (20 μM), and/or necrostatin-1 (necro, 40 μM) for 48 hours. Cell death was measured. Values represent means ± SD from three independent experiments. *: p<0.05, ***: p = 0.001.

Since AMDE-1 modulated autophagy at both the induction and degradation phase and it affected the lysosome function significantly we hypothesized that AMDE-1 could be explored as a chemotherapeutic agent for cancer therapy. In many cases, it has been shown that the toxicity of a chemotherapeutic agent could be enhanced by CQ since cancer cells can often mount a protective autophagy response toward the chemotherapeutic agent [17]. Being a dual modulation agent, AMDE-1 alone may serve this purpose. Indeed, AMDE-1 induced a dose-dependent cell death in HeLa cells in 48 hours (Fig 5A). The relative EC_50_ was calculated to be 1.1 μM. In contrast, the relative EC_50_ of CQ in the same type of cells was 27.5 μM, or about 25-fold higher (Fig 5B). Thus the toxicity of CQ at a low dose would become obvious only when in combination with other chemotherapeutic agents [19], whereas AMDE-1 at the same low dose seemed to be sufficient to cause significant cell death by itself. We compared the toxicity of the two agents in a number of other cancer cell lines, including a glioma cell line, U251; a colon cancer cell line, HCT116 and an erythroleukemia cell line K562 (Fig 5C). In each of these cases, 2.5 μM of AMDE-1 caused the same level of cell death as 50 μM of CQ, confirming that AMDE-1 was much more potent than CQ. Interestingly both agents could kill Atg5-deficient MEFs, though at a relatively lower rate, compared to the rate for some of the cancer cells. This result indicated that CQ and AMDE-1’s toxicity could be mediated by the lysosome-toxic effect without involving autophagy activation.

Both CQ and AMDE-1 can be toxic to non-transformed immortalized cells, such as MEFs and CCD-18Co cells, which are colonic cells (Fig 5C). It is worth to note that at the same effective doses AMDE-1 was less toxic than CQ to the non-transformed CCD-18Co cells and wild type MEFs, suggesting that AMDE-1 might have a better selectivity toward cancer cells. To further investigate this possibility, we compared the dose responses of HCT116 colon cancer cells and CCD-18Co cells to AMDE-1. Indeed, AMDE-1 induced a higher level of cytotoxicity in HCT116 cells than in CCD-18Co cells (Fig 5D). While the relative EC_50_ of AMDE-1 for HCT116 was 1.3 μm, that for CCD18-Co cells was 2.6 μm, or one-fold higher. When the dose response was normalized for the maximal response (100%), the absolute EC_50_ of AMDE-1 in HCT116 was 1.9 μm and that in CCD18-Co cells was 12.8 μm, or about 5.7-fold higher. Thus AMDE-1 seemed to be more selectively toxic to the transformed tumor cells.

To determine the mode of cell death induced by AMDE-1we added z-VAD-fmk, a pan-caspase inhibitor, or necrostatin-1, a RIP1 kinase inhibitor, to AMDE-1-treated cells. Necrostatin-1, but not z-VAD-fmk, was able to block AMDE-1 induced cell death in HCT116 cells (Fig 5E), suggesting that AMDE-1 induced necroptosis, but not apoptosis. Taken together, these results indicated that AMDE-1 could be explored as a cancer therapeutic agent with a potentially wide therapeutic window.

## Discussion

The discovery of useful small molecule modulators for autophagy is still challenging due to the complexity of the autophagic process. Several types of screening assays have been established that are based on a number of different parameters. Although by far the largest number of assays have used LC3 lipidation as the readout [6,7,8,9], assays based on PtdIns(3)*P* accumulation [6,7,8,9], Atg4 cleavage activity [3,5], and lysosome degradation [32] have also been developed. In addition, a functional screening was also successful in identifying chemicals that affect lipophagy [4]. The diversity of the screening platforms leads to the discovery of many different chemicals that can directly or indirectly affect the readout and therefore autophagy function. It is thus important to carefully dissect the potential mechanisms involved in each of these compounds to determine whether they can serve as chemical probes in research or have the potential for future therapeutic applications.

**Fig 6:**
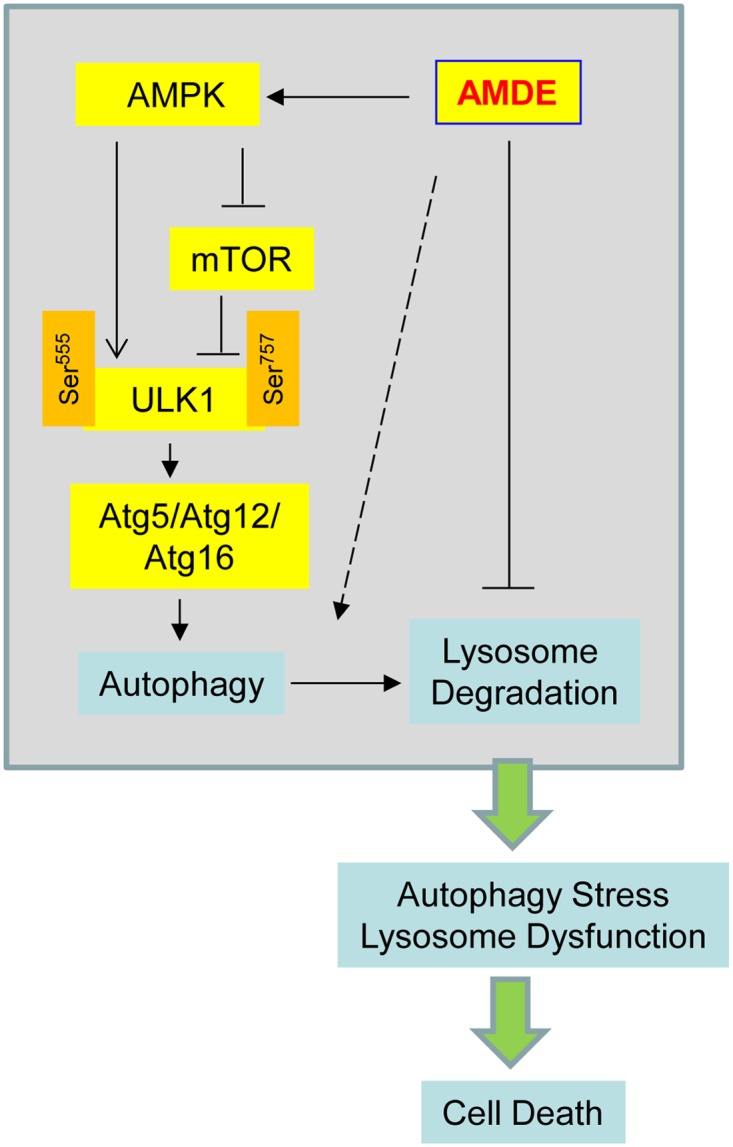
A proposed mode for the action of AMDE-1. AMDE-1 can trigger autophagy by the AMPK-mTOR-ULK1 pathway. However, AMDE-1 can also suppress lysosomal function and thus autophagic flux. Consequently, the lysosome dysfunction and/or the accumulation of non-degraded cellular material can promote cell death. The dashed arrow suggests that AMDE-1 may also affect autophagy by other unknown mechanisms.

AMDE-1 is a unique autophagy modulator that emanated from a high throughput screen of 196,160 compounds for LC3-GFP accumulation in punctate structures. Chemicals can affect GFL-LC3 distribution in a number of ways. After excluding non-specific effects, the increase or decrease in GFP-LC3 puncta could be attributed to either changes in autophagy activation or changes in autophagic degradation. Thus positives from the screen could be either autophagy inducers or inhibitors. Using a series of detailed mechanistic studies, we found that AMDE-1 represents a mechanistically unique compound that can exert both effects, but at different stages. Acting as an autophagy activator at the initiation stage and an autophagy inhibitor at the degradation stage causes a significant increase in GFP-LC3 puncta due to the failure to degrade the accumulated autophagosomes (Fig 6).

mTORC1 is one of the key suppressor of autophagy, which is positively regulated by upstream signals such as growth factors and amino acids, but negatively regulated by AMPK [33]. mTORC1 and AMPK can phosphorylate ULK1 at different sites, leading to its inactivation or activation respectively [25,26]. AMDE-1 induced canonical autophagy in Atg5 and Atg16L dependent fashion. Furthermore, it activated AMPK and suppressed mTORC1, which affected the phosphorylation pattern of ULK1, leading to its activation to promote autophagy.

The inhibitory effect of AMDE-1 on autophagy degradation was demonstrated by the accumulation of p62/SQSTM1, long-lived proteins and autophagosomes (as measured by the tandem GFP-RFP construct), which is likely due to suppression of lysosome enzyme maturation and activity. On the other hand, it does not seem that AMDE-1 is able to alter the lysosome acidity. Several well-established lysosome inhibitors including CQ, BafA_1_ and AC act to reduce lysosome acidity. Chemicals such as E64D and pepstatin can inhibit lysosome activity and AMDE-1 seems to work in a similar way. Although we have demonstrated that AMDE-1 could suppress the activity of cathepsin B, but not cathepsin D, using the lysosomal fraction, we had not tested this effect using recombinant enzymes. In addition, the substrates used for assessing cathepsin B and D activity could be also hydrolyzed by cathepsin C, L, and E, respectively. So it is necessary in future studies to use a panel of purified recombinant cathepsins to examine whether they could be directly affected by AMDE-1. It is also possible that lysosome enzymes other than proteases could be affected by AMDE-1. Nevertheless the present study establishes that AMDE-1 inhibits lysosomal function by affecting the activity of lysosome enzymes, but not lysosomal acidity.

The suppression of lysosome function could have an unexpected effect on autophagy activation because a normal lysosome function is also required for mTORC1 activation [8,34]. We had found previously that prolonged lysosome inhibition will lead to down-regulation of mTORC1 and thus in turn activation of autophagy [8]. We cannot rule out that AMDE-1 could inhibit mTORC1 in this fashion, particularly at the later stage. However, AMDE-1 can inhibit mTORC1, independently of lysosome inhibition, via AMPK activation at an early time point. Lysosome inhibition by CQ, E64D and pepstatin A do not activate AMPK, indicating that the ability of AMDE-1 to activate AMPK is unique and is not dependent on lysosome suppression. AMPK can also activate ULK1 at Ser^555^ independently of mTORC1, and thus would be also independent of lysosome inhibition. Taken together, AMDE-1 can induce autophagy via early upstream signaling events, other than the possible later feedback from the lysosome inhibition. Certainly, we could not rule out that AMDE-1 could affect autophagy by additional unknown mechanisms.

Small molecules that can induce and inhibit autophagy have received considerable attention as they can be useful pharmacological probes to dissect the complex autophagy process and may be explored as potential therapeutic agents. Autophagy inducers such as rapamycin and CBZ have been used to alleviate the pathology of neurodegeneration [15] and alpha-1 anti-trypsin deficiency [16] in animal models. A clinical trial has recently been launched for the latter (NCT01379469). One of the major possible applications of autophagy inhibitors could be for cancer therapy. Being under increased metabolic pressure, cancer cells may be more susceptible to the suppression of autophagy than non-cancerous cells [35]. Many studies have shown an increased effectiveness in controlling cancer cell growth when autophagy inhibitors are used in combination with standard chemotherapy or radiation therapy [17]. Several clinical trials have already been launched [17] and the preliminary results seem to be promising [36]. Inhibitors of autophagy degradation that are most commonly used in these studies are chloroquine (used in research) or its derivative hydroxychloroquine (used in human trials) because it is an FDA-approved drug in clinical use.

It is reasonable to assume that compounds that enhance autophagy induction while simultaneously impairing lysosomal degradation function could be quite useful as standalone agents for cancer therapy (Fig 6). Such agents can be cytotoxic because they are able to cause accumulation of more autophagosomes that fail to be degraded. Indeed AMDE-1 alone is sufficient to cause significant cell death at a dose that would not be toxic for CQ. CQ would need an about 24 times more dose to be equivalently toxic. The toxicity is also in part relevant to the dysfunction of lysosome that affects other non-autophagy functions, such as endocytosis. This may explain that AMDE-1 is still toxic to Atg5-deficient MEFs where autophagy level would be minimal. Nevertheless, lysosome dysfunction in the presence of autophagy can be highly detrimental [37]. We noticed that AMDE-1 was less toxic to non-transformed cells, such as MEFs and a colon cell line, CCD-18Co, which is a desired feature of antineoplastic agents and may translate into therapeutic benefit. It is important to further test AMDE-1 in animal models for its potential use in cancer therapy.

In summary, we have identified a molecule that could represent a new genre of autophagy modulators with dual effects in autophagy initiation and degradation. This novel property may be explored for selective killing of cancer cells that have a higher demand on autophagy function.

## Supporting Information

S1 Fig Imaging algorithm for the quantification of GFP-LC3-positive autophagosomes. Mouse embryonic fibroblasts (MEFs) stably expressing GFP-LC3 (5,000/well) were cultured with thapsigargin (TG, 0.25 uM) for 18 hours. Accumulation of GFP-LC3 in autophagosomal membranes (bright spots) was induced. Cells were imaged on an Array Scan II using a 20X objective and an Omega XF100 dual-bandpass filter set at excitation/emission wavelengths of 350nm/461nm (Hoechst) and 484nm/515nm (GFP), respectively. Cells were identified by Hoechst 33342 staining and compartmentalized into nuclear (blue outline) and cytoplasmic (green outlines) areas. The algorithm was interactively modified to quantify vesicular objects located in the cytoplasm (traced in yellow).
(PDF)

S1 Table The fifty-six top hits from the high content screening for chemical modulators of GFP-LC3 punctation. (PDF)

S2 Table Summary of the secondary analysis of the top hits. (PDF)
