# Supplementary figures and images for: AMDE-1 Is a Dual Function Chemical for Autophagy Activation and Inhibition
Source: PLoS One. 2015 Apr 20;10(4):e0122083. doi: 10.1371/journal.pone.0122083 (PMC4403922; doi:10.1371/journal.pone.0122083)

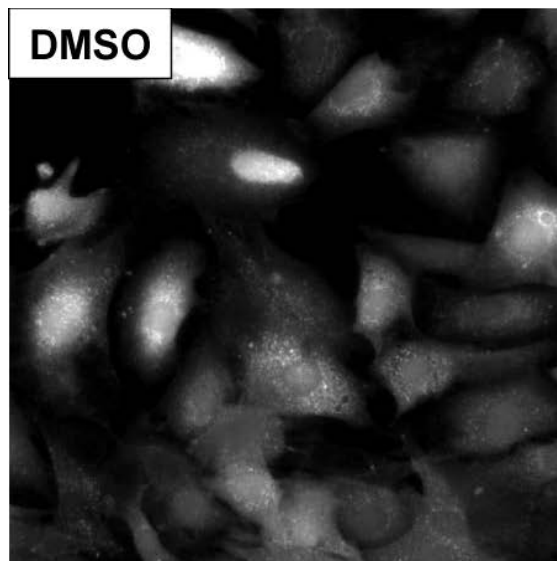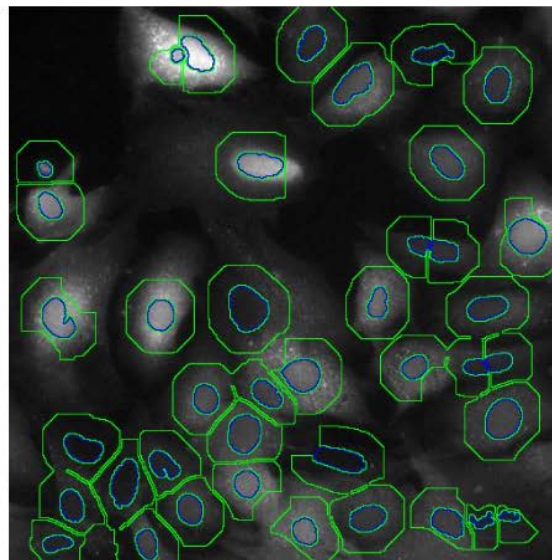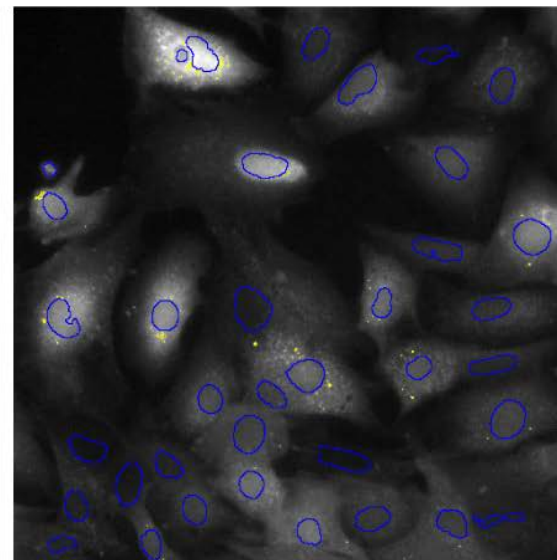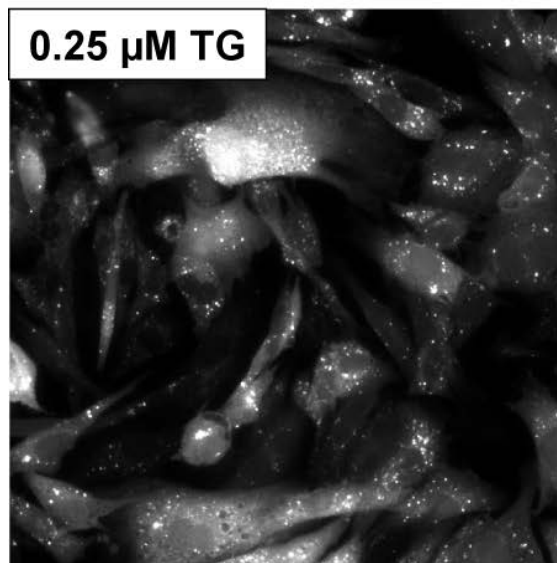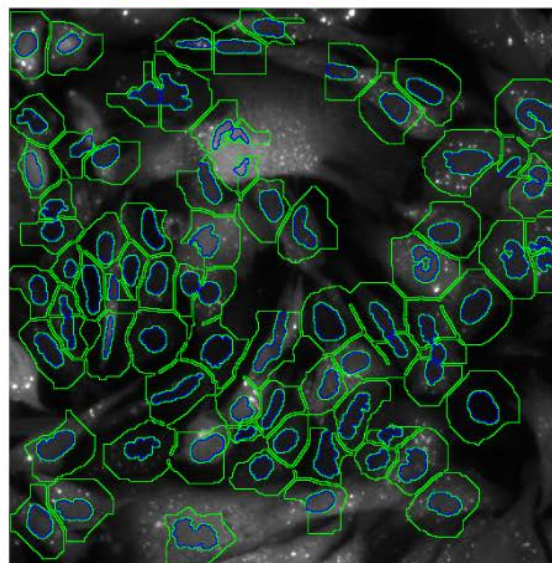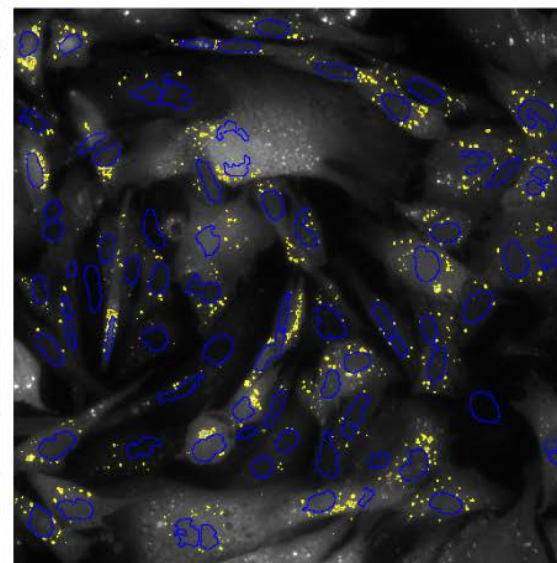

raw image

compartmentalize cells

detect and quantify spots

Supplement: S1 Fig — Mouse embryonic fibroblasts (MEFs) stably expressing GFP-LC3 (5,000/well) were cultured with thapsigargin (TG, 0.25 uM) for 18 hours. Accumulation of GFP-LC3 in autophagosomal membranes (bright spots) was induced. Cells were imaged on an Array Scan II using a 20X objective and an Omega XF100 dual-bandpass filter set at excitation/emission wavelengths of 350nm/461nm (Hoechst) and 484nm/515nm (GFP), respectively. Cells were identified by Hoechst 33342 staining and compartmentalized into nuclear (blue outline) and cytoplasmic (green outlines) areas. The algorithm was interactively modified to quantify vesicular objects located in the cytoplasm (traced in yellow). (PDF) [file pone.0122083.s001.pdf]
