# Supplementary material for: AMDE-1 Is a Dual Function Chemical for Autophagy Activation and Inhibition
Source: PLoS One. 2015 Apr 20;10(4):e0122083. doi: 10.1371/journal.pone.0122083 (PMC4403922; doi:10.1371/journal.pone.0122083)
Supplement: S1 Table — (PDF) [file pone.0122083.s002.pdf]

S1 Table. The fifty-six top hits from the high content screening for chemical modulators of GFP-LC3 punctation

PubChem SID	Compound ID	Well Ref	Average EC50 (microM)	SD	Cluster	Molecular Weight	Parent Atom Count	Hydrogen Bond Donors	Hydrogen Bond Acceptors	Lipinski Score	Polar Surface Area	ALogP	Rotatable Bonds
7975488	UPPDI-00086635	H1	0.66	0.21	Cluster 1 14736275	335.4	23	1	4	0	81.7	2.59	6
14727439	UPPDI-00182069	D2	0.98	0.14	Cluster 1 14736275	439.9	30	0	5	0	81.62	4.92	3
14736275	UPPDI-00164549	B2	0.76	0.39	Cluster 1 14736275	409.5	29	1	4	0	85.61	4.91	5
14745130	UPPDI-00160901	I1	0.8	0.41	Cluster 2 17408254	280.3	21	1	2	0	49.41	2.9	2
17408254	UPPDI-00417904	B3	5.29	0.3	Cluster 2 17408254	373.4	26	3	3	0	96.53	2.91	6
17387264	UPPDI-00172772	M2	3.39	1.97	Cluster 3 17507614	352.4	25	1	3	0	68.29	4.61	7
17507614	UPPDI-00273769	J3	5.08	3.1	Cluster 3 17507614	420.5	28	2	4	0	93.73	3.54	11
14724612	UPPDI-00432845	C2	0.46	0.2	Cluster 4 14741431	359.2	22	2	2	0	70.14	3.05	5
14733973	UPPDI-00442806	H2	0.23	0.11	Cluster 4 14741431	359.2	22	2	2	0	70.14	3.05	5
14741431	UPPDI-00437670	G2	0.95	0.39	Cluster 4 14741431	294.4	22	2	2	0	70.14	2.72	5
17386500	UPPDI-00402676	F2	0.63	0.33	Cluster 4 14741431	382.4	29	1	4	0	80.38	4.71	6
4260391	UPPDI-00423207	J1	1.53	0.69	Cluster 5 4262411	289.3	20	1	3	0	59.16	3.08	3
4262411	UPPDI-00433011	K1	0.4	0.25	Cluster 5 4262411	283.3	21	1	3	0	59.16	3.02	3
7967417	UPPDI-00417347	D3	4.86	1.62	Cluster 5 4262411	371.4	27	1	5	0	77.62	3.82	6
14737096	UPPDI-00438402	C1	0.41	0.27	Cluster 5 4262411	317.4	22	1	3	0	59.16	3.86	5
24786176	UPPDI-00442790	F4	1.59	1.11	Cluster 5 4262411	347.4	24	1	4	0	68.39	3.49	7
4246481	UPPDI-00046818	I3	2.84	0.23	Cluster 6 4254597	406.4	30	2	4	0	121.1	3.49	9
4254597	UPPDI-00026363	M1	0.1	0.08	Cluster 6 4254597	392.4	29	2	4	0	138.2	2.09	7
865858	UPPDI-00235089	O2	2.87	0.18	Cluster 7 7975000	479.6	36	1	4	1	69.99	5.1	7
7975000	UPPDI-00074596	L3	4.36	0.91	Cluster 7 7975000	446.5	34	1	3	1	70.66	6.56	6

24792287	UPPDI-00312614	A4	3.07	2.73	Cluster 8 24805246	391.2	24	0	4	0	81.35	3.42	4
24805246	UPPDI-00309295	N3	2.24	1.08	Cluster 8 24805246	346.8	24	0	4	0	81.35	3.32	4
24808048	UPPDI-00300765	O3	3.76	1.75	Cluster 9 24808160	295.3	22	0	3	0	46.61	2.91	4
24808160	UPPDI-00302197	C4	4.68	1.16	Cluster 9 24808160	295.3	22	0	3	0	46.61	2.91	4
844574	UPPDI-00038031	F3	3.34	1.18	Singletons	371.4	26	1	2	0	86.48	3.55	6
855810	UPPDI-00431229	K3	2.86	0.33	Singletons	280.4	20	2	3	0	66.76	1.96	0
856344	UPPDI-00423347	A1	0.42	0.09	Singletons	353.4	26	2	4	0	112.8	2.51	5
856923	UPPDI-00093356	G3	0.59	0.24	Singletons	376.4	26	1	5	0	84.94	2.63	6
3715743	UPPDI-00442368	E3	3.66	1.56	Singletons	374.9	26	1	3	0	50.8	3.89	6
4242320	UPPDI-00441807	A2	2.84	1.08	Singletons	388.4	28	1	5	0	78.38	2.66	8
4246416	UPPDI-00241543	J2	4.86	0.43	Singletons	320.3	24	0	4	0	52.83	2.85	3
4248717	UPPDI-00441806	L1	2.21	0.88	Singletons	386.8	26	2	3	0	64.88	3.11	2
7968184	UPPDI-00249910	H3	3.67	2.49	Singletons	415.2	26	1	4	0	73.06	4	3
14722047	UPPDI-00178695	B1	1.5	0.3	Singletons	378.5	28	2	4	0	70.25	4.6	5
14723293	UPPDI-00165759	N1	0.82	0.4	Singletons	341.5	24	0	2	0	37.38	4.69	2
14726587	UPPDI-00169864	A3	3.67	0.94	Singletons	418.5	30	2	2	0	101.8	3.6	8
14729169	UPPDI-00164382	E1	0.08	0.04	Singletons	446.3	28	0	5	0	109.5	3.45	6
14730495	UPPDI-00402826	G1	1.62	0.41	Singletons	415.7	28	0	3	1	49.57	5.98	5
14731704	UPPDI-00420366	P2	3.77	1.39	Singletons	349.4	26	1	5	0	94.02	4.15	5
14733320	UPPDI-00434762	L2	5.6	1.27	Singletons	392.4	27	0	1	0	23.55	4.75	3
14737900	UPPDI-00166878	P1	0.31	0.28	Singletons	354.5	25	1	3	0	50.69	4.45	7
14741249	UPPDI-00225071	E2	0.43	0.19	Singletons	474.6	32	1	5	0	74.24	3.72	9
14741499	UPPDI-00225330	O1	2.43	1.23	Singletons	316.4	24	0	2	0	43.6	4.84	5

14742376	UPPDI-00424732	D1	0.7	0.17	Singletons	370.5	27	1	2	0	76.84	4.95	4
14744172	UPPDI-00437212	K2	4.77	3.46	Singletons	380.3	25	1	2	0	32.7	4.56	7
14746058	UPDDI-00160238	F1	1.8	0.74	Singletons	247.3	18	2	4	0	76.24	2.31	6
17403673	UPPDI-00433648	C3	1.27	0	Singletons	380.4	29	1	3	1	50.69	5.04	7
17408907	UPDDI-00223742	I2	5.55	0.2	Singletons	364.8	25	0	3	1	53.75	5.25	6
17409719	UPDDI-00221798	N2	3.23	0.71	Singletons	371.3	24	0	2	1	36.68	6.33	4
17507447	UPDDI-00273503	M3	2.62	0.11	Singletons	219.3	15	0	2	0	29.54	2.27	2
24782220	UPPDI-00442793	G4	0.27	0.09	Singletons	335.4	25	1	4	0	58.89	4.75	4
24791273	UPPDI-00442779	E4	2.29	0.31	Singletons	249.3	18	3	3	0	100.2	0.85	5
24791537	UPDDI-00299547	B4	5.27	0.33	Singletons	330.2	24	1	8	1	188.6	2.82	3
24807884	UPPDI-00442787	H4	5.53	1.38	Singletons	446.5	33	3	6	0	116.7	3.25	8
24813857	UPDDI-00314756	D4	6.11	1.26	Singletons	472.6	35	1	3	0	57.58	3.97	10
24826599	UPDDI-00315071	P3	5.72	0.52	Singletons	277.7	19	0	3	0	31.23	3.52	4

The top hits from the high content screening assay were sorted first based on clusters and then based on the SID number. Listed here are also the compound ID of the University of Pittsburgh Drug Discovery Institute (UPDDI) and the well reference number for the confirmation study. The molecular weight and the chemical properties were also listed. SID 855810 (in purple) is brefeldin A. SID 14730495 (in red) is AMDE-1, which is featured in this study.
