# Supplementary material for: AMDE-1 Is a Dual Function Chemical for Autophagy Activation and Inhibition
Source: PLoS One. 2015 Apr 20;10(4):e0122083. doi: 10.1371/journal.pone.0122083 (PMC4403922; doi:10.1371/journal.pone.0122083)
Supplement: S2 Table — (PDF) [file pone.0122083.s003.pdf]

S2 Table. Summary of the secondary analysis of the top hits

SID	Cluster	Average EC50 (microM)	EC50 SD	1st-MEF-11h	1st-MEF-15h	1st-MEF-18h	2nd-MEF-18 hr	3rd-MEF-18 hr	Atg5KO MEF-18hr	A549-22 hr	Saos2-22 hr	WB on GFP-LC3-II (MEF)	Note
7975488	Cluster 1 14736275	0.66	0.21	7.51	8.51	13.3		18.6	0.8			Yes (+++)	
14727439	Cluster 1 14736275	0.98	0.14	2.51	5.03	13							
14736275	Cluster 1 14736275	0.76	0.39	5.45	5.52	10.7		22.7	3.3			Yes (+++)	
14745130	Cluster 2 17408254	0.8	0.41	4.66	9.77	17.3	17.3			16.2	4.3		
17408254	Cluster 2 17408254	5.29	0.3	91.2	92.8								Auto-fluorescent
17387264	Cluster 3 17507614	3.39	1.97	15.8	14.7	23.8	14.3			41.4	1.9		
17507614	Cluster 3 17507614	5.08	3.1	91.2	95.9		90.9	90.9	27.2	89.3	41.1	Yes (++)	
14724612	Cluster 4 14741431	0.46	0.2	6.7	4	6.21		39.2	5.4			Yes (++)	
14733973	Cluster 4 14741431	0.23	0.11	7.02	13.2	7.48							
14741431	Cluster 4 14741431	0.95	0.39	2.78	8.76	15.4							
17386500	Cluster 4 14741431	0.63	0.33	4.08	6.47	8.89							
4260391	Cluster 5 4262411	1.53	0.69	6.19	5.29	10.9							
4262411	Cluster 5 4262411	0.4	0.25	3.98	6.5	9.17							
7967417	Cluster 5 4262411	4.86	1.62	8.12	15		15	15	1.6	40	3.1	Yes (+++)	
14737096	Cluster 5 4262411	0.41	0.27	3.08	7.51	3.37							
24786176	Cluster 5 4262411	1.59	1.11	4.19	8.75								
4246481	Cluster 6 4254597	2.84	0.23	85.6	95.8		80.2	84.7	25.6	95.2	74.4	Yes (+++)	
4254597	Cluster 6 4254597	0.1	0.08	4.38	11.1	16.1	16.1	36.4	3.2	32.7	3	Yes (++)	
865858	Cluster 7 7975000	2.87	0.18	13.2	21.7	9.16	15.7	16.1	0.5	93.6	5.3	Yes (-/+)	
7975000	Cluster 7 7975000	4.36	0.91	43.5	70		70	70.0	26.5	87.1	66.7	Yes (+)	

24792287	Cluster 8 24805246	3.07	2.73	5.11	14.6		14.6	16	3.2	34.7	5.6	Yes (++)	
24805246	Cluster 8 24805246	2.24	1.08	9.32	16.2		16.2	17	4.1	26.7	2.2	Yes (++)	
24808048	Cluster 9 24808160	3.76	1.75	5.62	5.78								
24808160	Cluster 9 24808160	4.68	1.16	5.47	6.78								
844574	Singletons	3.34	1.18	40.9	62.1		62.1	29.9	0.3	73.4	21.4	Yes (+/-)	
855810	Singletons	2.86	0.33	81.2	86.5		86.5			44.2	54.1	Yes (+)	brefeldin A
856344	Singletons	0.42	0.09	6.79	4.71	11.5		37.8	1.67			Yes (+++)	
856923	Singletons	0.59	0.24	12.7	11.6								
3715743	Singletons	3.66	1.56	8.33	2.99								
4242320	Singletons	2.84	1.08	6.37	11.3	13.2							
4246416	Singletons	4.86	0.43	17.7	16.9	37.3	37.3			97.9	53.3	Yes (+)	
4248717	Singletons	2.21	0.88	4.93	5.63	4.17							
7968184	Singletons	3.67	2.49	14.8	14.9		14.9	14	1.6	34.9	3.7	Yes (+++)	
14722047	Singletons	1.5	0.3	3.35	6.42	11.7							
14723293	Singletons	0.82	0.4	6.29	8.06	7.14		21.6	2.1			Yes (+/-)	
14726587	Singletons	3.67	0.94	9.58	6.51								
14729169	Singletons	0.08	0.04	9.76	10.9	13.8							Toxic
14730495	Singletons	1.62	0.41	96.7	100	100	95	88.5	2.8	96.6	98.7	Yes (+++)	AMDE-1
14731704	Singletons	3.77	1.39	3.48	3.28	2.56							
14733320	Singletons	5.6	1.27	2.16	2.66	35.5							Auto-fluorescent
14737900	Singletons	0.31	0.28		13.8	11							
14741249	Singletons	0.43	0.19	91.2	95								Auto-fluorescent
14741499	Singletons	2.43	1.23	18.8	8.41	11.3							Auto-fluorescent
14742376	Singletons	0.7	0.17	2.22	3.51	3.85		11	2.7			Yes (+/-)	
14744172	Singletons	4.77	3.46	2.56	4.59	6.22							
14746058	Singletons	1.8	0.74	5.76	13.8	15.4	15.4			45.7	8.3		
17403673	Singletons	1.27	0	8.42	7.77								
17408907	Singletons	5.55	0.2	2.4	1.82	3.91							
17409719	Singletons	3.23	0.71	1.86	4.17	6.19							
17507447	Singletons	2.62	0.11										Cell Shrinkage
24782220	Singletons	0.27	0.09	8	13.8		13.8			46.2	3		
24791273	Singletons	2.29	0.31	7.73	14.7		14.7			43.8	4.1		

24791537	Singletons	5.27	0.33	95	100		91.8	84.4	25.7	93.2	88.7	Yes (+)	
24807884	Singletons	5.53	1.38	5.15	31.3		31.3	31.6	8.0	84	45	Yes (++)	
24813857	Singletons	6.11	1.26	61.7	91.3		91.3	77.2	16.7	93.8	35	Yes (+/-)	
24826599	Singletons	5.72	0.52										Cell Shrinkage
Medium				1.5			3.6	3.5	0.3	14.2	2.7	No	Negative Control
TG				90	95	95	98.5	97	39	88.5	35	Yes (+)	Positive Control

The compounds are listed in the same order as that in Supplemental Table 1. EC50 is derived from the primary screening and is the same as that listed in Supplemental Table 1. Secondary assays include manual analysis using MEFs (three times, 1st, 2nd and 3rd), A549 and Saos2 cells expressing GFP-LC3 at different times (11h to 22 hr) as indicated. Concentration of the compounds analyzed was 10 μM. The numbers shown are percentage of cells with 5 or more GFP-LC3 puncta. Secondary assays also include western blot analysis of GFP-LC3-II, which is the lipidated form of GFP-LC3, in MEFs treated with the indicated compounds for 16 hr. The intensity of the GFP-LC3-II was semi-quantified. Thapasigagin (TG, 1 μM) was used as a positive control. No entry indicates not-done. Compounds that are autofluorescent were determined using MEFs with no expression of GFP-LC3.

SID 855810 (in purple) is brefeldin A. SID 14730495 (in red) is AMDE-1, which is featured in this study.
